# Individual differences in online research: Comparing lab-based and online administration of a psycholinguistic battery of linguistic and domain-general skills

**DOI:** 10.3758/s13428-024-02533-x

**Published:** 2024-12-19

**Authors:** Kyla McConnell, Florian Hintz, Antje S. Meyer

**Affiliations:** 1 Max Planck Institute for Psycholinguistics, Wundtlaan 1, 6525 XD Nijmegen, The Netherlands; 2 Donders Centre for Brain, Cognition, and Behavior, Radboud University, Nijmegen, The Netherlands; 3 Philipps University, Marburg, Germany; 4 Center for Mind, Brain and Behavior, Philipps University Marburg & Justus Liebig University, Giessen, Germany

**Keywords:** Individual differences, Online experimentation, Psycholinguistics, Domain-general skills, Language skills

## Abstract

Experimental psychologists and psycholinguists increasingly turn to online research for data collection due to the ease of sampling many diverse participants in parallel. Online research has shown promising validity and consistency, but is it suitable for all paradigms? Specifically, is it reliable enough for individual differences research? The current paper reports performance on 15 tasks from a psycholinguistic individual differences battery, including timed and untimed assessments of linguistic abilities, as well as domain-general skills. From a demographically homogenous sample of young Dutch people, 149 participants participated in the lab study, and 515 participated online. Our results indicate that there is no reason to assume that participants tested online will underperform compared to lab-based testing, though they highlight the importance of motivation and the potential for external help (e.g., through looking up answers) online. Overall, we conclude that there is reason for optimism in the future of online research into individual differences.

## Introduction

Over the last several years, experimental psychologists and psycholinguists have begun to explore the potential of conducting studies online, outside of controlled laboratory environments. In particular, the development towards increased online research was accelerated by the restrictions on lab-based work during the height of the COVID-19 pandemic, which forced many researchers to explore whether an online lab could be brought to the participants’ homes. Researchers quickly began to share resources about best practices as well as technical and practical recommendations for behavioral experimentation outside of the lab (Blake & Dąbrowska, 2024; Garcia et al., 2022; Grootswagers, 2020; Rodd, 2024; Sauter et al., 2020).

However, do results collected online really compare with those collected in laboratory environments? Large, stable, and group-level effects seem to replicate well online. However, there is an increasing interest in psychology and psycholinguistics in differences between individuals and their causes (Dąbrowska, 2012; Engelhardt et al., 2017; Isbilen et al., 2022; Kidd et al., 2018, 2023; McConnell, 2023; McConnell & Blumenthal-Dramé, 2021; Payne et al., 2014; Pronk et al., 2022). Yet, paradigms of individual differences may face unique challenges when moved to an online lab. One important concern is that establishing individual differences requires precise estimation at the participant level (Hedge et al., 2018). Noise that may be leveled out in factorial designs could lead to erroneous conclusions about individual performance, and of course, systematic differences in participant motivation or any other extraneous pressures on participants could drive or obscure effects. It is not necessarily the case that paradigms that produce sensible data at the group level under online data collection will also be reliable in an individual differences paradigm (Haines et al., 2020; Hedge et al., 2018).

To address the suitability of online data collection to individual differences research in psycholinguistics, we compared performance on an extensive individual differences battery completed by large, demographically similar samples in a controlled lab setting or online. Results suggest reason for optimism in the use of online data collection for measuring stable individual differences; however, the role of two important considerations (looking up answers and motivation level) arise as important factors to keep in mind.

### Online research: Potentials and pitfalls

Psycholinguistic labs exist for good reasons. They ensure that all participants are tested under identical conditions, which are optimized for the purpose of the experiment (e.g., in a quiet, well-lit room without much distraction), and that they all fully understand what they are meant to do. Some details hardly need to be considered in lab research, such as the time of day (which influences visual perception through lighting, as well as participant tiredness), but can vary widely when research is taken out of the lab (Dandurand et al., 2008).

Despite the less controlled nature of online testing, copious previous research has established its validity for experimental psychology. Many classic psychological effects replicate well under online data collection, including psychometric assessments. For instance, comparable performance has been found in measures of cognitive inhibition like the Stroop, Flanker, Simon, and go/no-go tasks, measures of memory like digit spans and two-back tasks, as well as visual search and attentional blink paradigms (Crump et al., 2013; Germine et al., 2012; Miller et al., 2018; Semmelmann & Weigelt, 2017). Challenging higher-order cognitive tasks such as face reading and memory for abstract art also pattern similarly in online compared to lab-based data (Germine et al., 2012). Standard psycholinguistic effects have also been replicated in online studies, including effects of word frequency, age of acquisition, and name agreement on the speed of picture naming, lexical decision, and self-paced reading, among others (Corps & Meyer, 2023; Fairs & Strijkers, 2021; He et al., 2021; Hilbig, 2016; Vogt et al., 2021).

Importantly, results are also similar when the same participants complete the same tasks both online and in the lab. Miller and colleagues (2018) invited 127 participants to complete multiple tasks assessing processing speed, including the go/no-go task, the two-back task, and a number-letter task, both in the lab and online, with 1 week between the tests in the two settings. A diffusion model showed no significant difference in central tendency, and the internal reliability of the test scores was similar for the two sessions. Slightly more variance (approximately 5%) was observed in online administration, however, and test–retest reliability between the two sessions was weak for some tasks (range: 0.33 to 0.73). Overall, the results strongly suggest data collected online is comparable to data collected in the lab. However, it is hard to say if this conclusion extends to individual differences designs because the data were analyzed at the group level.

Of course, collecting research data online is not without risk. Two primary issues can be identified: technical concerns related to the quality of the experimental software and the hardware available to the participants and issues related to participants’ behavior in a remote setting. The first of these issues has been addressed for many of the most common experimental platforms and programming environments (Anwyl-Irvine et al., 2021; Bridges et al., 2020; Reimers & Stewart, 2015) and is not considered here. Instead, we focus on the second issue, i.e., how participants’ behavior is affected by the testing environment. Specifically, we compare the scores of young Dutch students tested online or in the lab. A comparison of demographically more varied groups (e.g., students vs. online crowdsourced workers) is beyond the scope of the current paper (but see Hauser & Schwarz, 2016; Peer et al., 2021). In this, our study provides novel information about the way test scores may differ between relatively homogeneous, well-matched groups when tests are administered in a lab or online.

A major factor in participant behavior is likely to be motivation. In psycholinguistics, there is a growing appreciation for the role of motivation (or the lack thereof) in language tasks (Christianson et al., 2022). This includes the realization that “… some portion of published work [consists] of data that have been collected from unmotivated, uninterested, or disengaged participants”, even if it is collected in the lab (Christianson et al., 2022, p. 54). We might expect that participants tested online are less motivated or struggle to maintain an appropriate level of motivation for the duration of a study. In comparison, participants in the lab have already committed to coming to a new environment and are participating under the careful eye of an experimenter. Indeed, online data is often found to contain more variation, though the exact causes for this have not been identified (Miller et al., 2018; Semmelmann & Weigelt, 2017).

Motivation may affect the task level in that certain task types might demand more attention and focus than others. Difficult tasks see considerable drop-out rates in online experiments, though an engaging online test-taking environment can mitigate the difference in performance (Dandurand et al., 2008; Pedersen et al., 2023; Rodd, 2024). This may be particularly relevant to individual differences studies if drop-out is self-selecting; that is, if participants are more likely to withdraw from an online experiment if they find it particularly difficult (e.g., because they have a weakness in the tested skill or because they are less able to resist distraction). Drop-out is likely to be less severe in in-person settings because participants would have to excuse themselves from the room and leave the building.

Additionally, participants may not be willing or able to create conditions that would facilitate performance in online experimentation. For instance, only half of the participants in one online study reported doing the experiment in a quiet environment, although they agreed to participate while free of distraction (Simcox & Fiez, 2014). In another study, nearly a third of online participants reported watching TV while doing experimental tasks (Chandler et al., 2014). Applied to an individual differences context, participants who are more prone to distraction, less motivated, or otherwise less engaged may be especially likely to multitask, thus lowering their scores. Again, in the laboratory, these variables are the researcher’s responsibility and not within the participant’s control.

On the other hand, certain participants may strive too strongly for peak performance. Online participants have the opportunity to score higher than their in-lab counterparts in any task where they can easily look up answers. For some tasks, this is as easy as opening another browser window, glancing at one’s phone, or even asking a nearby friend for a second opinion. In one study, 10% of crowdsourced online participants correctly indicated the number of African countries, compared to none in a face-to-face sample (Goodman et al., 2013). Participants can possibly be discouraged from using external help by clear instructions and reassurance that they are not expected to answer every question correctly, but it is difficult to entirely eliminate such behavior (Goodman et al., 2013). Perhaps this is simply linked to modern life, where any information is merely a click away.

Importantly, a bulk of the previous research on the difference between research in the lab and on the web focuses on group-level differences. Yet online research is especially advantageous to individual differences work. Correlational research demands large sample sizes with sufficient within-sample variability, and these two aspects can be difficult to obtain through convenience sampling in the (university) population close to the lab. In contrast, online experiments have the potential to reach participants that are representative of the general public and many participants can be tested in parallel (Berinsky et al., 2012). In recent years, these benefits have attracted some of the first individual differences research to be conducted online, and the results have been comparable to what would be expected in the lab (Isbilen et al., 2022; McConnell & Blumenthal-Dramé, 2021; Vermeiren & Brysbaert, 2024; Vermeiren et al., 2022). Yet the questions raised about data quality, the ability to seek external help, and the role of motivation mean that comparing similar participants’ performance on the same tasks in the lab and online is still needed. To assess these questions, we compare data from fifteen linguistic and non-linguistic tasks collected either online or in the lab as part of the Individual Differences in Language Skills (IDLaS-NL) battery.

### IDLaS-NL: online and in the lab

The data from the IDLaS-NL battery is ideal for investigating the feasibility of individual differences research in an online environment. The battery was designed for young adult speakers of Dutch and includes 31 assessments of linguistic and non-linguistic skills (Hintz et al., 2023).^1^ The full test battery was completed online over four sessions by a sample of 579 young Dutch speakers. In addition, 169 participants completed the battery in a designated lab space under the supervision of an experimenter.

Online participants completed all 31 tasks of the battery using the experimental software *Frinex* (FRramework for INteractive Experiments (Withers, 2016). For inhouse testing, 15 tasks were implemented using *Frinex*, while the other 16 used the software *Presentation*, which is designed for laboratory and not online use. We compared only the fifteen tasks that both groups completed on *Frinex*: five untimed assessments of receptive linguistic skills or linguistic experience, three timed speech production tasks, four assessments of working memory and an assessment of nonverbal reasoning. These 15 tasks thus cover both linguistic knowledge and language production, as well as domain-general cognitive ability. In the following section, each of these tests will be described briefly and functionally; for details about the technical setup and the design of each task, the reader is directed to Hintz et al. (2020, 2024).^2^

This dataset has several advantages when considering the current question. First of all, the sample size is large, which affords strong statistical power. At the same time, the participants all come from the same, rather homogenous population: Dutch-speaking young people (primarily students) between the ages of 18 and 30. Further, we administered a large battery of tests from different domains of linguistic and non-linguistic ability. This allows us to assess the impact of online testing in general and the relative impact of the testing environment on different test types. For example, some types of assessments may be easier to look up the answers to, while others demand more motivation or focus on the part of the participant.

In summary, the IDLaS-NL dataset contains data from a large sample of demographically similar participants on several linguistic and domain-general aspects of individual differences. All participants completed the tasks in the same technological setup. In the following section, we outline the tasks involved in the battery, and look at their distributions under online or laboratory assessment. We also submit them to a hierarchical Bayesian model to ensure that the trends seen in the descriptive statistics are maintained. To establish that the patterns are not only maintained at the group level but are also sustained in the correlation patterns between the tests and the constructs they measure, we also assess measurement invariance between online and in-lab participants. With this methodology, we assess the effect of online testing on fifteen diverse tasks from the domains of language production, language comprehension, and domain-general processes. We ask how participant-based factors such as motivation and the potential to look up answers affect data collected online. Ultimately, we aim to determine whether online testing is suitable for individual differences designs across a range of tasks typical to behavioral psycholinguistics.

## Method

### Participants

One hundred sixty-nine participants completed the test battery in a designated psycholinguistic lab at the Max Planck Institute for Psycholinguistics in Nijmegen, The Netherlands. Participation was spread over 2 days, with two hour-long blocks on each day. Participants were tested individually or in groups of up to five people. Five hundred seventy-nine participants took part in the same tasks online, using a simulated version of a Chrome browser. Only the 15 tasks that were performed on the same experimental software (Frinex) are considered here. All participants gave written or digital informed consent to participate in the study. Permission to conduct the study was provided by the Ethics Board of the Social Sciences Faculty of Radboud University (application number: ECSW-2019–74). A full description of the lab setup can be found in Hintz et al. (2024).

In the current paper, we only consider participants that had usable scores for all fifteen tasks. In the lab sample, 20 participants did not have full datasets (12%) and in the online sample, 64 did not have full data sets (11%). This left data from 149 participants in the lab and 515 online. Table 1 shows which tasks contained missing values. Note that some participants were missing more than one task. Overall, we see that despite the fact that the percentage of participants without a full dataset is very similar in the two samples, there is more data missing from the language production tasks online than in the lab. In particular, for the maximum speech rate task (reciting the months of the year as fast as possible), this was often because participants mumbled or dropped syllables, leading to trials that could not be used. As for the verbal fluency tasks, scores were discarded when participants misunderstood the directions (e.g., by listing only incorrect words). This did not happen often in the lab, where participants could ask questions about the instructions.

**Table 1:** Number of participants missing a score by task and setting

	In-lab *N* = 169	Online *N* = 579
Antonym production	0	0
Idiom recognition	0	0
Spelling	0	1
Author recognition (DART)	3	1
Prescriptive grammar	0	0
Peabody (PPVT)	0	1
Digit span (forward)	0	2
Digit span (backward)	1	3
Corsi span (forward)	3	2
Corsi span (backward)	1	2
Nonverbal reasoning	3	0
Verbal fluency (semantic)	3	16
Verbal fluency (phonological)	1	17
RAN	3	6
Max speech rate	5	24

**Fig. 1:**
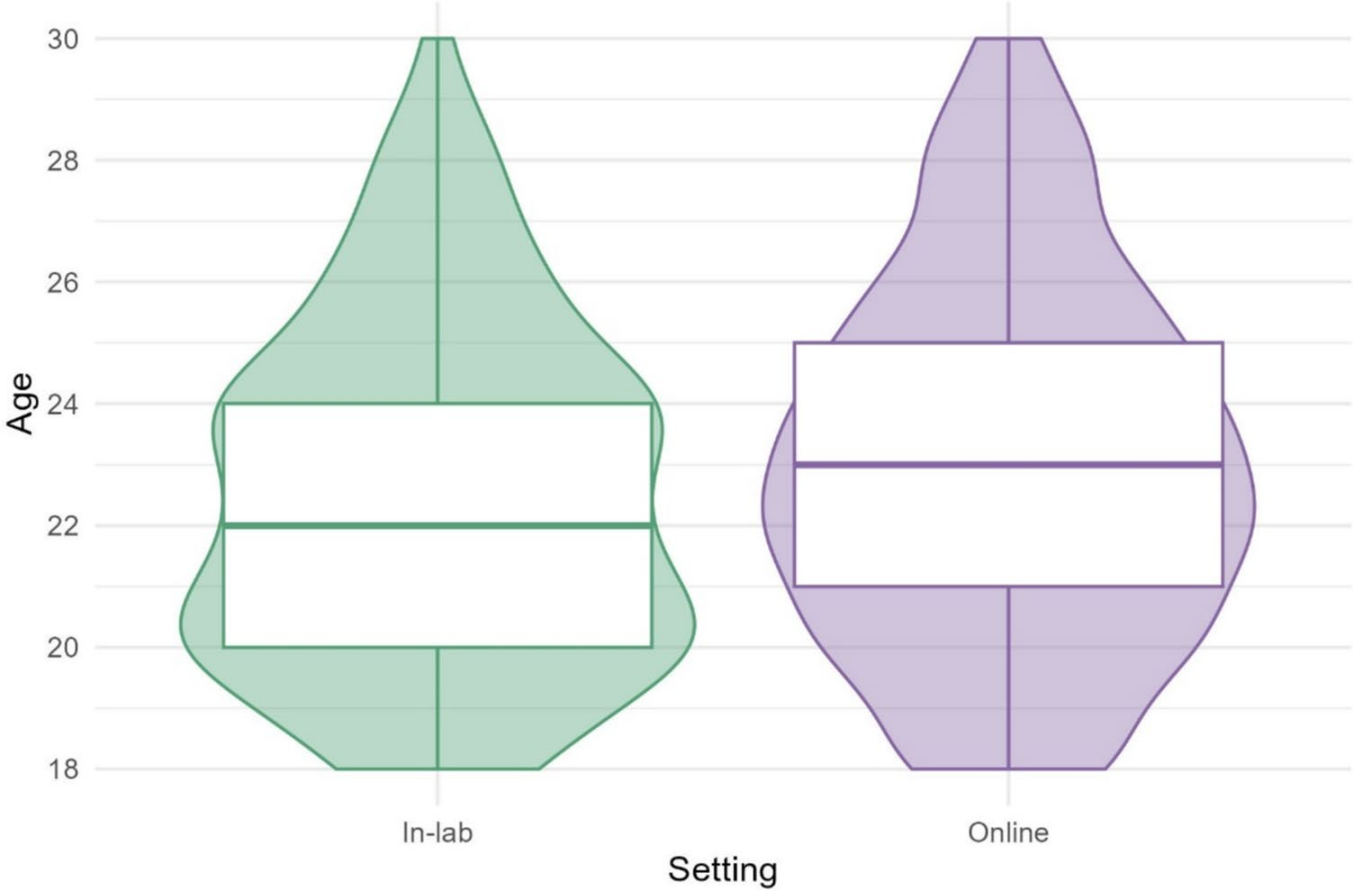
Participant ages in the lab and online, respectively. The boxplots show the median, interquartile range (IQR), and range and are overlaid on (mirrored) histograms to show the distribution of the data

Table 2 shows the number and percentage of participants based on gender, whether they were a student, and their level of education at the time of testing. The Dutch education system has been simplified here, so that the label “High school” includes the qualifications *HAVO*, *VWO*, *(V)MBO*, *Basisonderwijs*, and anyone who selected “other” (*N* = 5). Participant ages are visualized in Fig. 1. Although the values differ slightly, the samples are similar; all participants were Dutch young people between 18 and 30, primarily college-educated or in a university program. The most noticeable difference is that the online testing condition had more participants who identified as female (78.6% online compared to 53.7% in the lab). Although the age distributions vary slightly, the mean age in both settings is comparable (22.4 in the lab, 22.9 online).

**Table 2:** Demographic characteristics of in-lab and online participants

	In-lab (*N* = 149)	Online (*N* = 515)
Gender		
Female	80 (53.7%)	405 (78.6%)
Male	68 (45.6%)	108 (21.0%)
Highest level of education		
High school	88 (59.1%)	226 (43.9%)
Bachelor	48 (32.2%)	204 (39.6%)
Master	13 (8.7%)	85 (16.5%)
Student status		
Student (at time of testing)	119 (79.9%)	379 (73.6%)

### Untimed linguistic knowledge tasks

**Fig. 2:**
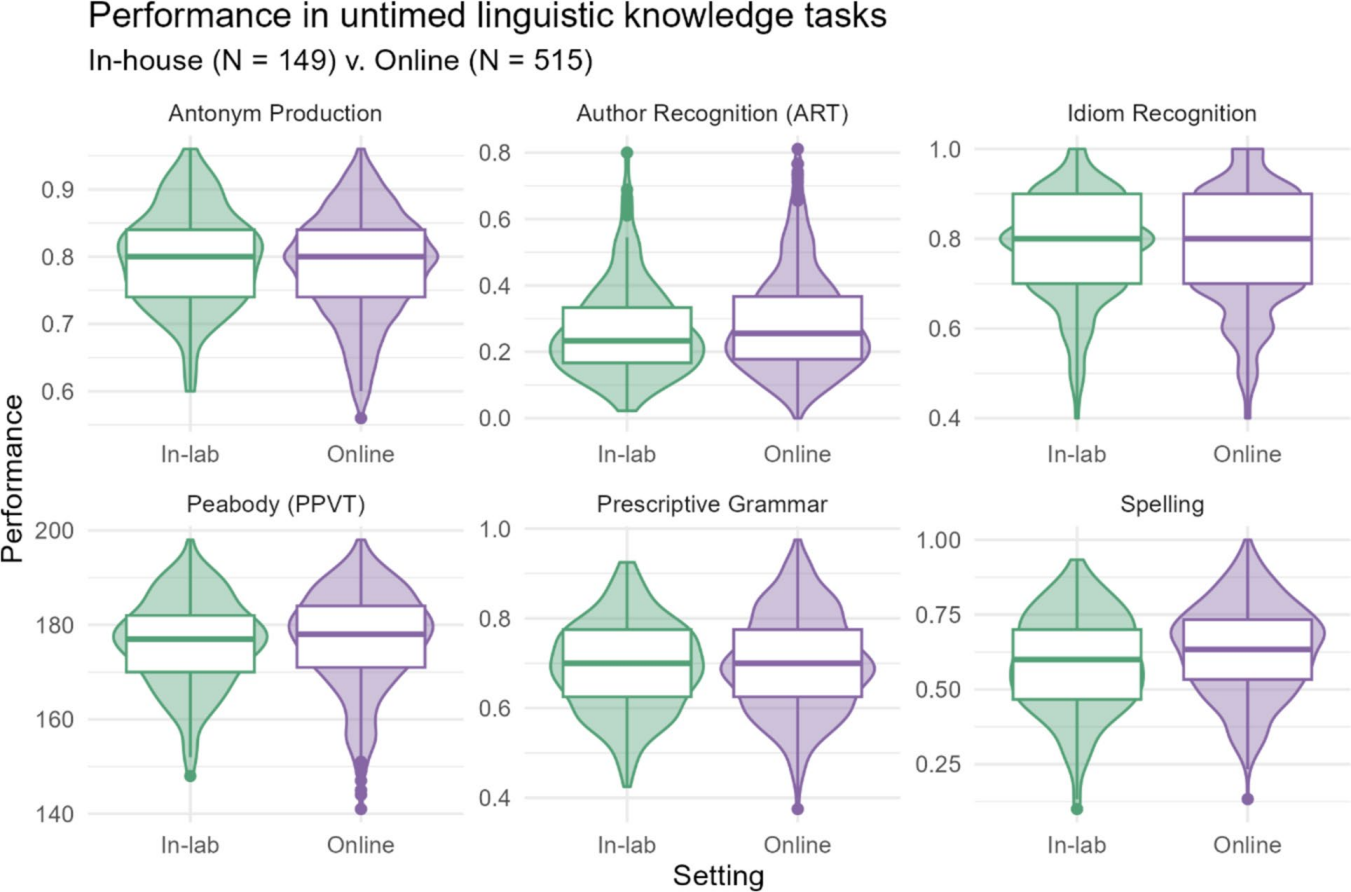
Distribution of performance in the untimed linguistic tasks, across in-lab and online samples. The boxplots show the median, interquartile range (IQR) and range and are overlaid on (mirrored) histograms to show the distribution of the data

The untimed linguistic tasks included in this analysis are an antonym production test, the Dutch Author Recognition Test (DART), an idiom recognition task, the Peabody Picture Vocabulary Test, a prescriptive grammar task, and a spelling task. All of these assessments tap into the participant’s linguistic knowledge or experience in an untimed test setting. Each test is described briefly in terms of the participant’s task below. The descriptive statistics for each sample for each test are shown in Table 3, and their distributions are visualized in Fig. 2.

**Table 3:** Descriptive statistics (mean, median, SD, min, and max) for all untimed linguistic tasks across in-lab and online samples

Untimed linguistic knowledge tasks					
Setting	Mean	Median	SD	Min	Max
Antonym production					
In-lab	0.80	0.80	0.07	0.60	0.96
Online	0.79	0.80	0.08	0.56	0.96
Author recognition (DART)					
In-lab	0.27	0.23	0.14	0.02	0.80
Online	0.29	0.26	0.15	0.00	0.81
Idiom recognition					
In-lab	0.78	0.80	0.11	0.40	1.00
Online	0.77	0.80	0.13	0.40	1.00
Peabody (PPVT)					
In-lab	176.10	177.00	9.32	148.00	198.00
Online	176.79	178.00	10.36	141.00	198.00
Prescriptive grammar					
In-lab	0.69	0.70	0.10	0.42	0.92
Online	0.70	0.70	0.11	0.38	0.98
Spelling					
In-lab	0.59	0.60	0.16	0.10	0.93
Online	0.63	0.63	0.15	0.13	1.00

#### Antonym production

For this task, participants both saw and heard a word, and then were asked to say the antonym to this word (Mainz et al., 2017). There were 25 trials. The task was not timed, and the participant’s score was the proportion of correctly produced antonyms.

#### Dutch Author Recognition Test (DART)

Participants completed the Dutch Author Recognition Test (DART) (Brysbaert et al., 2020), which is a common assessment of exposure to print language. For this, they saw 132 proper names, of which 90 were real authors and 42 were non-author foils. Their task was to identify which of the names were authors. A participant’s score was the proportion of correctly identified authors minus the proportion of ‘false alarms’ (foils that the participant indicated were authors).

#### Idiom recognition

Participants saw ten Dutch idioms selected from a normed database (Hubers et al., 2019). One idiom appeared on the screen at a time, together with four possible meanings; both idioms and meanings could be listened to by clicking on an icon. The task was to select the correct meaning for the idiom, and the score was calculated as the proportion of trials for which the correct meaning was selected.

#### Peabody Picture Vocabulary Test (PPVT)

Participants completed the online version of the standardized Dutch-language PPVT (third edition) (Dunn & Dunn, 1997; Schlichting, 2005). On each trial, they saw four drawings and heard a single word. Their task was to select which of the four pictures best matched the meaning of the word they heard. The assessment has a standard staircase procedure so that participants moved to a harder block (of 12 items) only if they performed well enough in the previous block; the test was started at the block recommended for people aged 18 to 35. A participant’s score was calculated, as recommended, as the number of the last item they responded to, minus the amount of errors they had made in the test.

#### Prescriptive grammar

For this task, participants heard 40 sentences and had to indicate whether they were grammatically correct. Half of the stimuli set featured correct Dutch sentences, while the other half had one of five common errors: the incorrect use of a personal pronoun (“ze”, they vs. “hun”, their; “ik”, I vs. “mij”, me), a comparative (“als”, as vs. “dan”, than), a relative pronoun (“die”, this vs. “dat”, that) and the participles of complex verbs (e.g., “stofgezogen”, vacuumed). A participant’s score was the proportion of correct responses.

#### Spelling

The spelling assessment contained 60 words frequently misspelled in Dutch. Half the items were spelled correctly and half represented common misspellings. Participants saw all words simultaneously and were asked to identify which words were misspelled. A participant’s score was calculated as the proportion of correctly identified misspelled words minus the proportion of ‘false alarms’ (words spelled correctly that the participant indicated were misspelled).

### Timed word production tasks

**Fig. 3:**
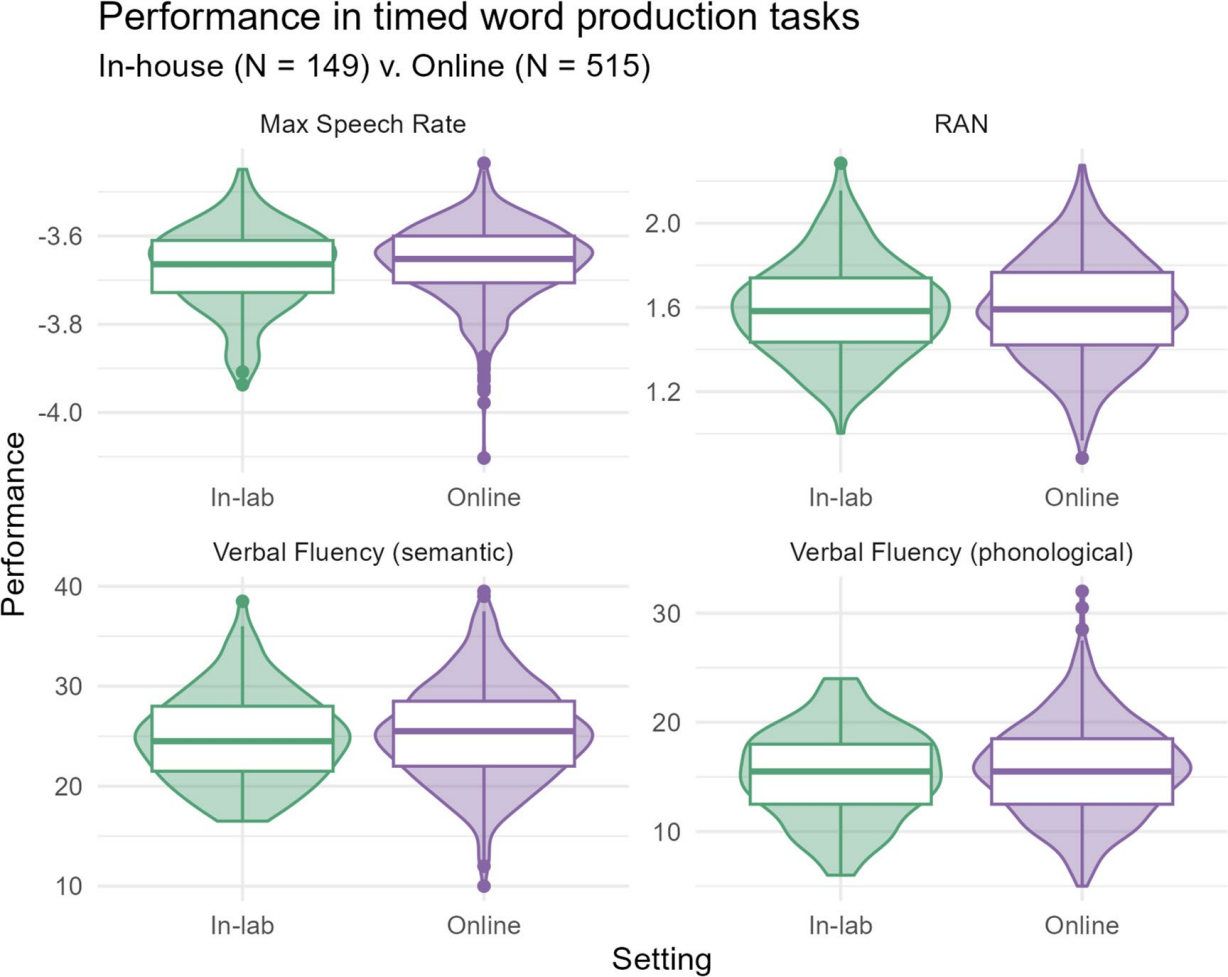
Distribution of performance in the timed word production tasks, across in-lab and online samples. The boxplots show the median, interquartile range (IQR), and range and are overlaid on (mirrored) histograms to show the distribution of the data

The second category of tasks we analyzed is the timed word production tasks. For this domain, we had two verbal fluency tasks, one of which was phonological and the other was semantic. We also included the maximum speech rate and rapid automatized naming (RAN). The tasks are described briefly, their descriptive statistics are listed in Table 4, and their distributions are shown in Fig. 3.

**Table 4:** Descriptive statistics (mean, median, SD, min and max) for all timed linguistic production tasks, across in-lab and online samples

Timed word production tasks					
Setting	Mean	Median	SD	Min	Max
Maximal speech rate					
In-lab	–3.67	–3.66	0.09	–3.94	–3.45
Online	–3.66	–3.65	0.09	–4.10	–3.44
Rapid automatized naming (RAN)					
In-lab	1.60	1.58	0.23	1.00	2.29
Online	1.59	1.59	0.24	0.88	2.27
Verbal fluency (semantic)					
In-lab	24.87	24.50	4.48	16.50	38.50
Online	25.36	25.50	4.89	10.00	39.50
Verbal fluency (phonological)					
In-lab	15.23	15.50	4.12	6.00	24.00
Online	15.76	15.50	4.35	5.00	32.00

#### Maximal speech rate

Participants were asked to name the months of the year as quickly as possible while maintaining good pronunciation. There were two identical trials. The score was calculated as the average duration (measured from utterance onset to offset) over both trials, if both were correct; otherwise, the duration of the correct trial was used. The duration was log-transformed and inversed.

#### Rapid Automatized Naming (RAN)

This test is a classic assessment of speeded word form access (Denckla & Rudel, 1976). Participants were familiarized with five line drawings and a name for each of these items, which was the most common name in a norming study (Araújo et al., 2021). They then saw all the drawings arranged in 5 × 6 grid and their task was to name all objects from left to right, starting at the top row and proceeding to the following rows. There were four sets featuring different pictures. Each set was used twice in different orders within the grid. The participant’s score was the ratio of the correctly named objects by the duration of speech (in seconds) during the trial, averaged over all sets.

#### Verbal fluency (semantic)

Participants were given a semantic category (for the first trial, “animals”, and for the second, “food and drink”). They were given 1 min to name as many words that fit the category as possible (Shao et al., 2014). Their score was the number of unique words produced in a minute, averaged over both categories.

#### Verbal fluency (phonological)

Participants were given a letter (for the first trial, “M”, and for the second, “S”). They were given 1 min to name as many words that started with that letter as possible (Shao et al., 2014). Their score was the number of unique words produced in a minute, averaged over both letters.

### Domain-general skills

**Fig. 4:**
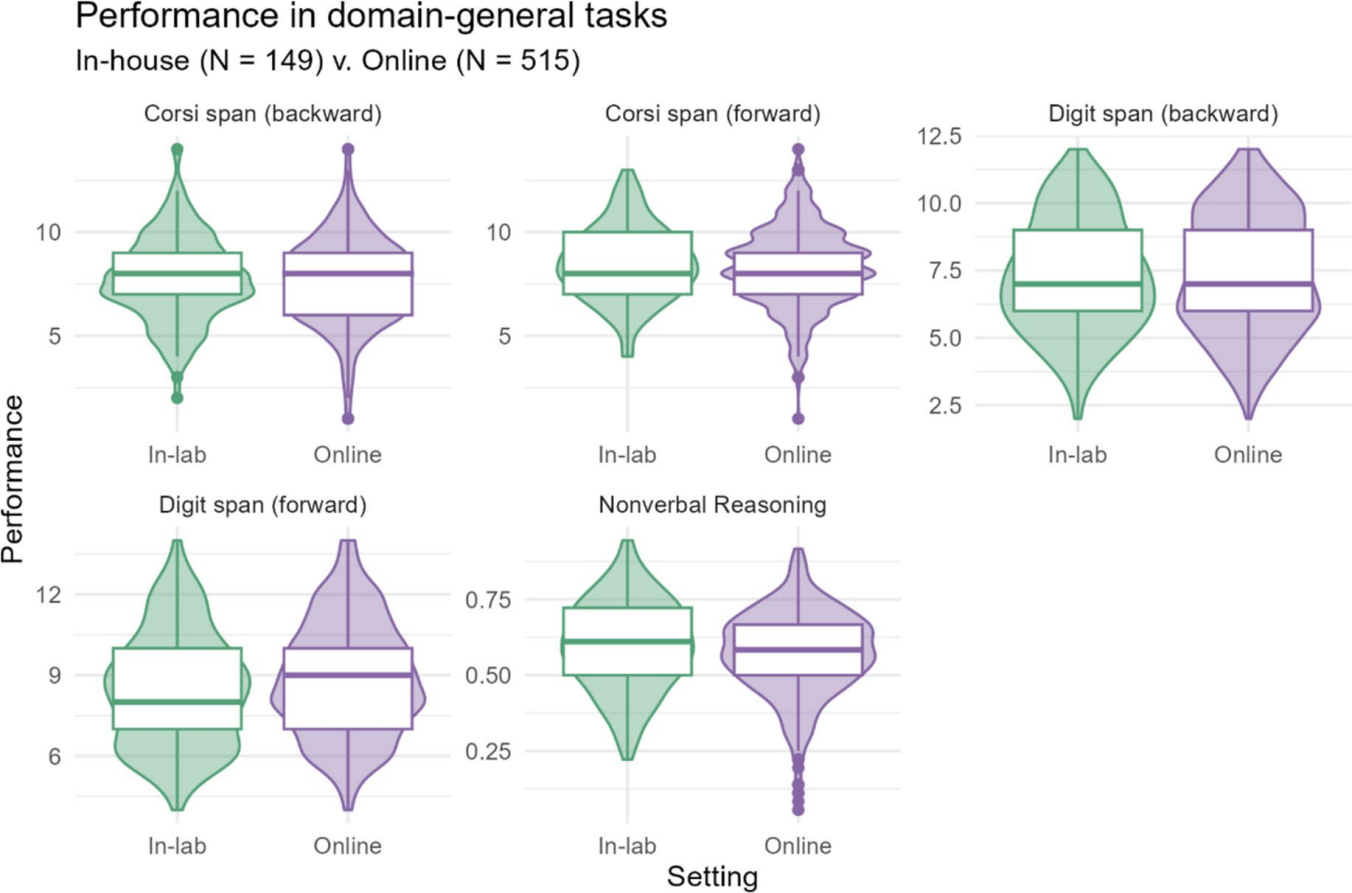
Distribution of performance in the domain-general tasks, across in-lab and online samples. The boxplots show the median, interquartile range (IQR) and range and are overlaid on (mirrored) histograms to show the distribution of the data

There were five assessments of domain-general skills, four of which tested working memory. The Corsi block clicking task measures visual working memory, while the digit span measures verbal working memory. We also assessed nonverbal reasoning via Raven’s advanced progressive matrices. The descriptive statistics for these tasks are listed in Table 5 and visualized in Fig. 4.

**Table 5:** Descriptive statistics (mean, median, SD, min, and max) for all domain-general tasks, across in-lab and online samples

Domain-general skills					
Setting	Mean	Median	SD	Min	Max
Corsi span (backward)					
In-lab	7.73	8.00	2.05	2.00	14.00
Online	7.65	8.00	2.05	1.00	14.00
Corsi span (forward)					
In-lab	8.48	8.00	1.77	4.00	13.00
Online	8.29	8.00	1.90	1.00	14.00
Digit span (backward)					
In-lab	7.28	7.00	2.09	2.00	12.00
Online	7.38	7.00	2.22	2.00	12.00
Digit span (forward)					
In-lab	8.48	8.00	2.10	4.00	14.00
Online	8.88	9.00	2.11	4.00	14.00
Non-verbal reasoning (Raven’s)					
In-lab	0.60	0.61	0.15	0.22	0.94
Online	0.57	0.58	0.14	0.06	0.92

#### Corsi span (forward and backward)

The Corsi block clicking test (Berch et al., 1998) assesses visuospatial short-term memory. Participants saw nine blocks on the screen, which lit up one by one at a speed of one square per second. Their task was to recall this sequence and re-create it after the prompt ended by clicking on the blocks. In the forward version, they clicked on the blocks in the same order as they saw them light up in the prompt phase. In the backward version, they clicked on them in the reverse order. Trials started at a length of three consecutive squares (after the practice items), and participants saw a longer sequence if they answered one of two trials of the same sequence length correctly. That is, if they correctly answered one of two trials in which three squares were lit, the next trials would have four squares light up. The assessment ended when two consecutive trials were answered incorrectly or at the end of the test (spans of nine squares). A participant’s score was the sum of correct responses over the version (forward or backward).

#### Digit span (forward and backward)

Participants heard spoken numbers and were asked to type the numbers they heard into a text box after auditory playback had finished (Wechsler, 2004). For the forward version, they typed numbers in the same order they had heard them, and for the backward version, in the opposite order (starting with the last number they heard). Similar to the Corsi span, trials started at 3 digits long (after the practice items), and participants heard a sequence that was longer by one digit if they answered two consecutive trials correctly. The assessment ended when two consecutive trials were answered incorrectly or at the end of the test (spans of 9 digits in the forward version, and 8 in the backward version). A participant’s score was the sum of correct responses over the version.

#### Nonverbal reasoning

Nonverbal reasoning was assessed using Raven’s Advanced Progressive Matrices test (Raven et al., 1998). For this, participants saw a cue and were asked to pick one of eight shapes/visual items that best completed the pattern shown in the cue. Participants could skip items and return to them later, or indicate that they did not know the answer. There were 36 items (excluding practice trials) and participants had 20 minutes to answer as many as possible. Their score was calculated as the proportion of trials they answered correctly.

## Results

Looking at the raw data and descriptive statistics, we can see that performance online was very similar to performance in the lab for the majority of tasks. Both the summary and the distributions visualized with violin and box plots are also highly comparable. Nonetheless, performance across the two groups was also assessed in a Bayesian mixed model. This was a useful inferential tool because the question at hand revolves around support for the null hypothesis (rather than, for example, submitting the data to a frequentist model, which cannot support the null, just quantify evidence against it.) Further, the by-subject random intercepts control for any systematic by-participant variance that may differ between the two groups. We describe the model parameters and output in the following section.

### Preprocessing

In the current analysis, we compare 15 tasks on very different scales; some are measures of accuracy in proportion to the total trials, others represent the number of words recalled, or the duration of articulation. In order to compare performance on diverse tasks, we assess a participant’s performance on a task in relationship to the mean and the standard deviation of scores across all participants in the task. For this, we calculated *z*-scores by task, so that a value of 0 represents the mean performance in the given task, and a value of 1 or −1 represents performance one standard deviation above or below the mean, respectively. This *z*-score served as the dependent variable for the models described below.

### Bayesian mixed effects model

To statistically evaluate the role of setting (in-lab or online) of each of the tasks, we fit a Bayesian mixed model predicting a participant’s performance by the interaction between task and setting and a random intercept by participant, using the R package *brms* (Bürkner, 2017). A main effect of task was not predicted, since the dependent variable was *z*-scored by task (i.e., the mean of every task was 0, and the standard deviation (SD) was 1). A main effect of setting was also not included because it would have represented the average effect across all fifteen tasks, not all of which we assumed to have the same relationship to setting (i.e., some tasks may have higher scores online where others have lower scores). Therefore, the main effect of setting would not have been interpretable in any meaningful way. Thus, the model formula was always: Performance ~ Task: Setting + (1|Participant). Both Setting (two levels) and Task (14 levels) were sum coded to facilitate the interpretation of the interaction term, and are therefore referenced to the grand mean across all levels.

Priors were centered at 0, since we could expect setting and task to affect the scores either positively or negatively. Because the DV is a *z*-score with mean 0 and SD 1, the prior for the intercept was set to be quite certain around 0 (a normal distribution with mean 0 and SD 0.5), and we set the same prior for the residual error sigma, which is automatically trimmed by brms to not allow values below zero. Because the design was optimized to capture differences between individuals, we set a slightly wide prior for the by-participant random intercept, a normal distribution with mean 0 and SD 1.

For the interaction of task and setting, we set three different priors, so that we could later check that the prior wasn’t exerting undue pressure on the results. For this, we fit:

- a “wide” prior – a normal distribution with mean 0 and SD 1;
- a “moderate” prior – a normal distribution with mean 0 and SD 0.5;
- and a “narrow” prior – a normal distribution with mean 0 and SD 0.2.

Priors were investigated visually before model fitting to ensure that they were reasonable in scale. We focus primarily on the model with “moderate” priors; the results for the other two models are very similar, and are available together with the full analysis script and data: https://osf.io/2knzc/

Looking at the numeric model output in Table 6, we first see that the intercept is, as expected, estimated at 0.00. Most of the tasks differ by Setting to only a very minimal degree, and all credible intervals cross 0. The by-setting estimates should be interpreted as the difference between the grand mean and the setting, not the raw difference between the two settings. All estimates of the interaction between task and setting have credible intervals that contain 0, showing that the true effect cannot be distinguished from 0. This indicates that there is no evidence for differences across the two types of test administration.

**Table 6:** Model output from Bayesian mixed model predicting *z*-score performance by the interaction between Task and Setting, from the model with a “moderate” prior for beta

Task	Setting	Estimate	Std. Error	CI (low)	CI (high)
Intercept	NA	0.0032	0.0946	– 0.1809	0.1892
By-participant random intercept	NA	0.4260	0.0155	0.3964	0.4569
Residual by-item variance	NA	0.9193	0.0068	0.9061	0.9327
Antonym production	In-lab	0.0770	0.1185	– 0.1562	0.3066
Antonym production	Online	– 0.0845	0.1014	– 0.2817	0.1126
Author recognition	In-lab	– 0.0789	0.1187	– 0.3115	0.1538
Author recognition	Online	0.0742	0.1019	– 0.1252	0.2714
Digit span (backward)	In-lab	– 0.0244	0.1189	– 0.2588	0.2066
Digit span (backward)	Online	0.0196	0.1019	– 0.1812	0.2173
Digit span (forward)	In-lab	– 0.0948	0.1180	– 0.3257	0.1356
Digit span (forward)	Online	0.0905	0.1022	– 0.1102	0.2894
Idiom recognition	In-lab	0.0232	0.1187	– 0.2096	0.2560
Idiom recognition	Online	– 0.0296	0.1020	– 0.2292	0.1686
Max speech rate	In-lab	– 0.0642	0.1184	– 0.2949	0.1670
Max speech rate	Online	0.0594	0.1019	– 0.1405	0.2571
Nonverbal reasoning	In-lab	0.0848	0.1191	– 0.1507	0.3183
Nonverbal reasoning	Online	– 0.0915	0.1021	– 0.2925	0.1050
Picture vocabulary	In-lab	– 0.0366	0.1189	– 0.2718	0.1938
Picture vocabulary	Online	0.0308	0.1022	– 0.1713	0.2282
Prescriptive grammar	In-lab	– 0.0385	0.1184	– 0.2706	0.1932
Prescriptive grammar	Online	0.0335	0.1017	– 0.1664	0.2303
Rapid automatized naming	In-lab	0.0020	0.1184	– 0.2299	0.2361
Rapid automatized naming	Online	– 0.0076	0.1021	– 0.2092	0.1888
Spelling	In-lab	– 0.1426	0.1184	– 0.3778	0.0871
Spelling	Online	0.1390	0.1015	– 0.0605	0.3347
Verbal fluency (phon)	In-lab	– 0.0636	0.1192	– 0.2950	0.1701
Verbal fluency (phon)	Online	0.0576	0.1021	– 0.1437	0.2551
Verbal fluency (sem)	In-lab	– 0.0537	0.1183	– 0.2874	0.1751
Verbal fluency (sem)	Online	0.0490	0.1023	– 0.1513	0.2467
Visual span (forward)	In-lab	0.0174	0.1185	– 0.2141	0.2475
Visual span (forward)	Online	– 0.0237	0.1023	– 0.2236	0.1744
Visual span (backward)	In-lab	0.0472	0.1184	– 0.1869	0.2798
Visual span (backward)	Online	– 0.0538	0.1022	– 0.2544	0.1430

Despite this, there are a few tasks worth mentioning. In the Spelling task, participants online have better scores (β = 0.139 [– 0.0605, 0.3347]) compared to in the lab (β = – 0.1426 [– 0.3778, 0.0871]). Scores for the forward digit span are also better online (β = 0.0905 [– 0.1102, 0.2894]), though the scores from the backward digit span does not show the same pattern (β = 0.0196 [– 0.1812, 0.2173]). On the other hand, for Nonverbal Reasoning, participants in-lab have better scores (β = 0.0848 [– 0.1507, 0.3183]) compared to online (β = – 0.0915 [– 0.2925, 0.105]). Antonym Production also shows a similar pattern, in that participants in the lab perform better (β = 0.077 [– 0.1562, 0.3066]) compared to online (β = – 0.0845 [– 0.2817, 0.1126]). However, credible intervals for all of these terms cross 0.

**Fig. 5:**
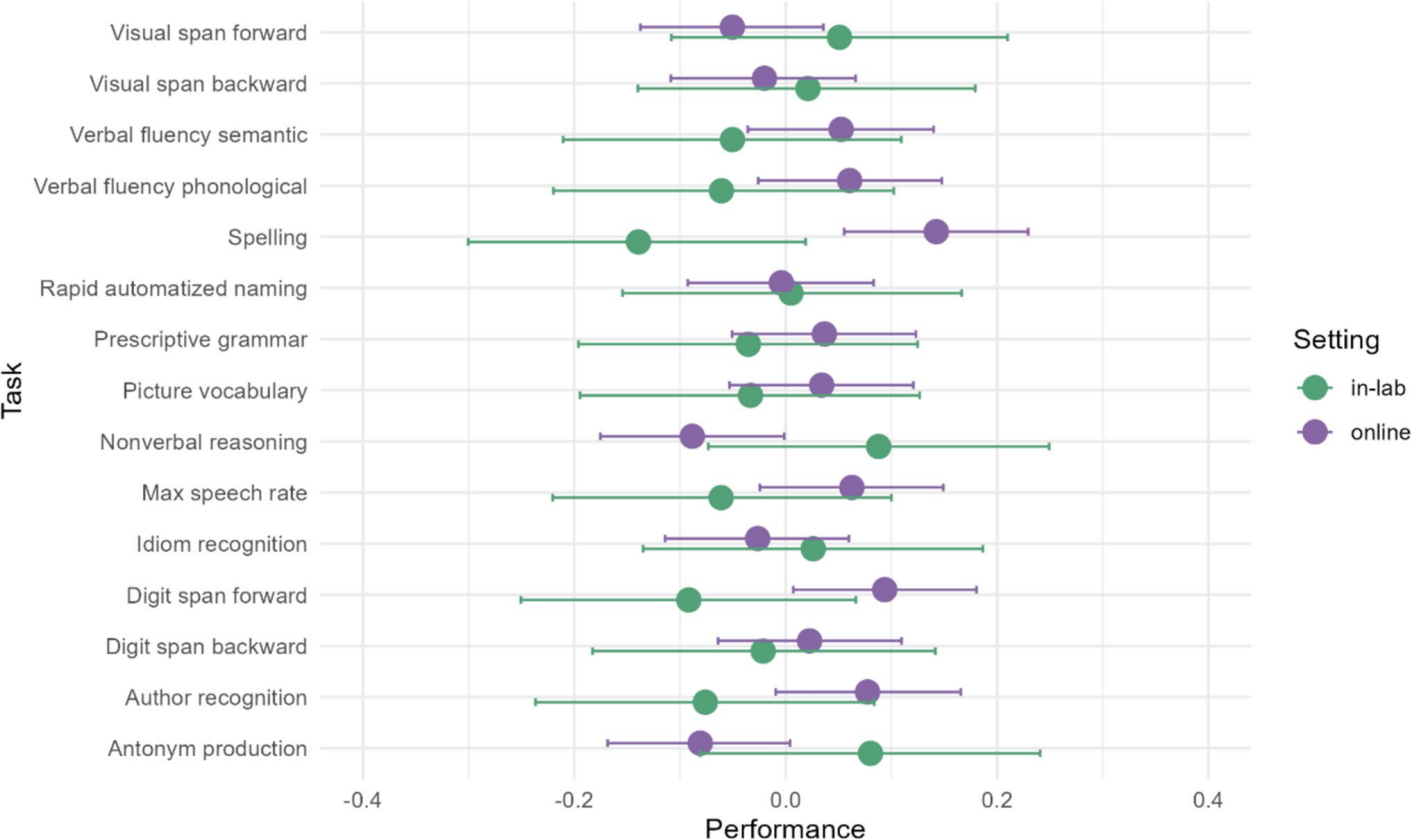
Conditional effects of the interaction between Setting and Task, based on the model with “moderate” priors, reported in Table 5. The points show the model predictions for the given combination of task and setting and the bars visualize the credible intervals, as generated by brms::conditional_effects (Bürkner, 2017). Note that the dependent variable is a task-dependent *z*-score; the value 0 is relative to the mean of the particular task

We also see that participants vary significantly compared to each other. The estimate for by-participant variation is 0.426 [0.3964, 0.4569]. There is also considerable by-item variation, resulting in residual error (estimate: 0.9193 [0.9061, 0.9327]); this is difficult to interpret, however, as the data is from different tests. The conditional effects are visualized in Fig. 5. The figure makes clear that the estimates for the in-lab setting are less exact, because this group is smaller. We also see that the by-setting estimates for the spelling task do not overlap, unlike those for all other tasks.

### Sample size

Individual differences research necessitates large datasets, particularly in the number of participants. Nonetheless, it may not be feasible (or necessary) for every project to sample from more than 500 online participants, as done here. Additionally, the data we compare have unequal group sizes; there are only 149 participants in the lab. Perhaps there is more variability in the online participants’ performances than in the in-person participants’ performances and this is being obscured by the fact that there is simply more data from the online group. To assess whether a model with balanced sample sizes across the two settings would lead to the same conclusions as the model reported above, we ran the model on a subset of the data with 149 participants from each setting. Participants were selected randomly without repetition from the full sample.

**Fig. 6:**
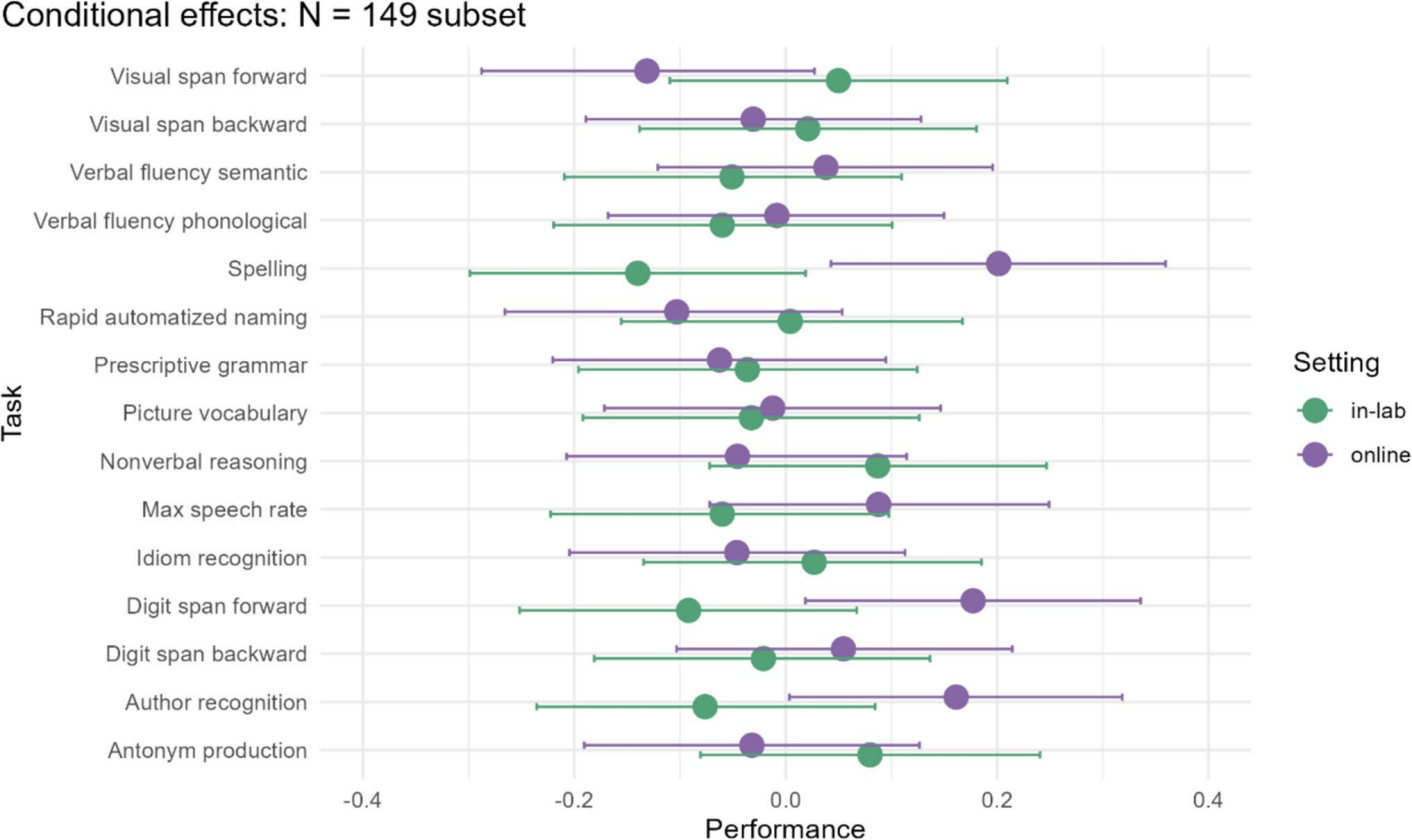
Conditional effects of the interaction between Setting and Task, for a random subset of 149 participants from each setting. The points show the model predictions for the given combination of task and setting, and the bars visualize the credible intervals, as generated by brms::conditional effects (Bürkner, 2017)

The full model output is reported in the OSF repository for this project: https://osf.io/2knzc/. Here we report the conditional effects for an easy visual overview of the results in this subset. Figure 6 shows the model predictions. With less data, the predictions for the online group now include more uncertainty. The general pattern of effects seen in Fig. 5 is still preserved. One notable but minor difference is that the difference between performance in the Antonym Production task appears less drastic in this subset due to the slightly lower prediction and broader credible intervals. However, the credible intervals for all tasks in the online setting are now wider, and this may be simply an artifact of the random subset that was selected.

### Measurement invariance

Establishing that the performance in the 15 tasks is similar across settings is important evidence for the feasibility of conducting psycholinguistic individual differences research online. However, a further important consideration is whether the tests measure the underlying constructs (untimed linguistic knowledge, timed word production, and domain-general ability) in the same way regardless of whether the tasks were administered online or in the lab. For this, we need to establish measurement invariance (Meredith, 1993). Measurement invariance is established if the association between test scores and the constructs they measure is not affected by group membership, so that participants who have the same underlying ability in the construct are expected to receive similar test scores, regardless of setting (Schmitt & Kuljanin, 2008; Van De Schoot et al., 2015).

To test for measurement invariance across the two settings, we followed the procedure outlined by Van De Schoot et al. (2012). This involves fitting a confirmatory factor analysis (CFA) model to both groups separately then comparing the fit to progressively more constrained confirmatory factor analyses. Specifically, we first fit a model constrained on factor loadings only; this means that both groups are set to have the same loadings from test scores onto the overarching construct that the test is intended to measure. If this model shows a similar fit, we can claim to have *metric invariance*. In the next step, we compare this model with one that has the same factor loadings and the same intercepts (the “zero point” for each test). If this more constrained model does not show a worse fit, we can speak of *scalar invariance*. Finally, we can compare this model to one in which factor loadings, intercepts and residual variance is constrained to be equal across both groups. If this model does not show a worse fit, we have *strict invariance*. If the models show worse fit in any of the steps, we can allow individual test scores to have different factor loadings, intercepts, and residual variances in the two groups. If this is necessary, we can continue to test for measurement invariance, but then have *partial measurement invariance*, indicating that some of the tests have different psychometric properties in the two groups.

**Fig. 7:**
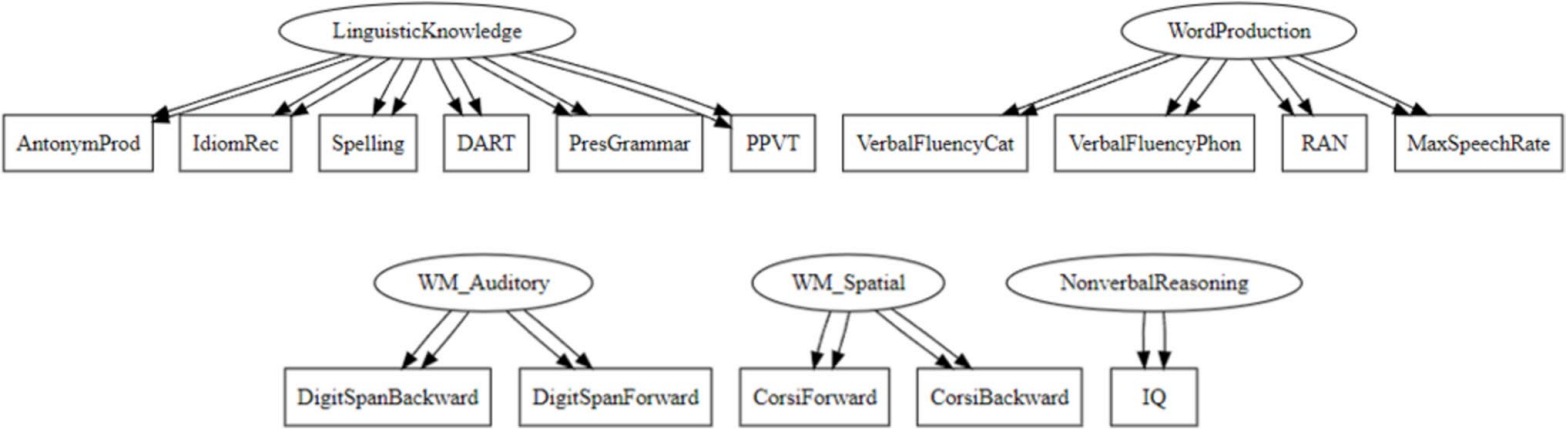
Graphical representation of the factor structure used to compute measurement invariance. The *double arrows* indicate the two settings (in-lab and online participants). The plot was generated with lavaanPlot (Lishinski, 2024)

In a first step, we fit a multigroup confirmatory factor analysis (CFA) using lavaan (Rosseel, 2012) based on the constructs of untimed linguistic knowledge, timed word production, and domain-general skills. Data from all participants in both groups were included. The first two constructs (Linguistic Knowledge and Word Production) included the tasks in Tables 3 and 4, respectively. For domain-general skills, we defined three sub-constructs: auditory working memory (including the digit span tasks), spatial working memory (including the Corsi block clicking tasks), and non-verbal reasoning (including the Raven’s matrices task). The model specification is visualized in Fig. 7. In this initial model (Model 1), each setting could vary in factor loadings, intercepts and residual variance, but the overall structure of the model (the constructs) was held constant. This model fit the data well (Comparative Fit Index (CFI) = 0.924, Tucker–Lewis index (TLI) = 0.902 root mean square error of approximation (RMSEA) = 0.052),^3^ indicating that the factor structure we specified was well represented by the data.

After establishing that the configural structure of the two groups was equivalent, we compared nested models in a stepwise fashion, constraining the differences between the two groups (in-lab and online participants). First, the factor loadings were constrained to be equal across both settings (Model 2). This did not cause a significant decrease in model fit, as shown with a chi-square model comparison test (*p* = 0.5392) and a comparison of the difference in fit as indexed by the alternative fit indices CFI (0.001), TLI (0.006) and RMSEA (– 0.002) for the constrained compared to the less constrained model.^4^ This indicates metric invariance, which we can interpret to mean that the tests are measuring the constructs at the same scale in both groups, or that the tests load onto the constructs in the same way regardless of setting (Putnick & Bornstein, 2016).

In the next step, both the factor loadings and the intercepts were constrained to be equal across groups. The aim was to establish scalar invariance: that the constructs are measured at the same scale and with the same zero point. In this, we assess whether the differences between the test scores in the two settings are driven by different underlying abilities in the two groups, not by a difference in the way that the tests are able to capture these differences based on the setting. The model with constrained factor loadings and intercepts (Model 3) showed a worse fit to the data in the chi-square model comparison, although it did not show a worse fit in the fit indices (*p* = 0.001769, CFI difference = – 0.009, TLI difference = – 0.006, RMSEA difference = 0.002). This could be due to the relative sensitivity of a chi-square test to small differences in large sample sizes (Chen, 2007).

A closer look showed that the parameters from two tasks were driving this effect: Spelling and Antonym Production. First, we allowed just the Spelling test to vary between the two groups, since this is the test that showed the largest difference in the model output. This model (Model 4) showed an improved fit, but the χ^2^ test still showed a preferential fit for Model 2, for which intercepts were not constrained for any task (χ^2^ *p* = 0.03164, CFI difference = – 0.001, TLI difference = 0.004, RMSEA difference =  < 0.001). Allowing both the Spelling and the Antonym Production tasks to vary (in Model 5) resulted in a comparable fit to Model 2 (χ^2^ *p* = 0.3603, CFI difference = – 0.005, TLI difference = – 0.001, RMSEA difference = – 0.001). Fit statistics for all models are reported in Table 7. The fact that the fit was worse when constraining the intercepts in both groups to be the same, unless the Spelling and Antonym Production tasks were allowed to freely vary between the settings, suggests that these two tests measure untimed linguistic knowledge differently based on the setting they are presented in (in-lab or online). However, partial scalar invariance has still been achieved.

**Table 7:** Comparison of fit for all nested models by Comparative Fit Index, Tucker–Lewis index and root mean square error of approximation

Model	Description	CFI	TLI	RMSEA
Model 1	Multigroup CFA	0.924	0.902	0.052 [0.043, 0.061]
Model 2	+ equal loadings	0.925	0.908	0.050 [0.041, 0.059]
Model 3	+ equal loadings and intercepts	0.915	0.902	0.052 [0.043, 0.060]
Model 4	+ equal loadings and intercepts (except Spelling)	0.920	0.907	0.050 [0.042, 0.059]
Model 5	+ equal loadings and intercepts (except Spelling & Antonym)	0.924	0.912	0.049 [0.040, 0.058]
Model 6	+ equal loadings, intercepts, and residuals (except Spelling & Antonym)	0.921	0.914	0.048 [0.040, 0.057]

Still allowing for free parameterization of the Spelling and Antonym Production tasks by group, in a final step we compared a model that had constrained factor loadings, intercepts and residual variance by group (Model 6). Constraining the residual variance is a necessary step for establishing strict measurement invariance. The model with these constraints did not show a worse fit (χ^2^ *p* = 0.1245, CFI difference = – 0.003, TLI difference = 0.003, RMSEA difference = – 0.001), indicating that measurement error and residual variance were also similar across groups (Putnick & Bornstein, 2016). With this, we establish strict (partial) measurement invariance for these tasks in regards to setting (in-house or online participation). We return to a consideration of these results in the Discussion. Full analysis scripts for measurement invariance are included in the OSF repository associated with this paper, along with the full model outputs: https://osf.io/2knzc.

## Discussion

Collecting data online is increasingly popular in psycholinguistics and psychology in general. Moving data collection online can facilitate experiments with groups that are either geographically dispersed or otherwise less likely to come into a research lab or university. Participants can join the experiment on their own time and data can be collected in parallel without the need for direct supervision of multiple testing sessions. The ease and pragmatism that comes with this type of data collection is accompanied by promising results. Data collected online from many of the most common psychological and linguistic paradigms confirm that results from participants tested online are comparable to those from in-lab samples (Crump et al., 2013; Germine et al., 2012; Hilbig, 2016; Miller et al., 2018; Patterson & Nicklin, 2023; Semmelmann & Weigelt, 2017). The advantages of online testing are particularly relevant for individual differences studies, which are growing in popularity (Engelhardt et al., 2017; Isbilen et al., 2022; Kidd et al., 2018, 2023; McConnell, 2023; McConnell & Blumenthal-Dramé, 2021; Payne et al., 2014). Importantly, however, individual differences paradigms rely on stable estimation of participant ability and thus must detect what group-level designs can safely level out, and it is unclear if research collected online can meet this standard (Hedge et al., 2018).

The Individual Differences in Language Skills (IDLaS-NL) dataset is unique in that it tests samples from the same population (young Dutch-speaking adults under the age of 30, largely in the university context) in the lab and online (Hintz et al., 2020, 2023). It also contains multiple tasks from different domains of linguistic and domain-general skills. Assessing the same population over diverse tasks is perfect for exploring the effect of online testing. We take the subset of this dataset that was collected on the same software for participants in the lab and online, which consists of fifteen tests from three domains: untimed linguistic knowledge, timed word production and domain-general ability (in the form of working memory and nonverbal reasoning).

We first looked at the descriptive differences between the two settings by comparing the means, medians and standard deviations, as well as the minimum and maximum scores for each task in each condition. These look exceptionally similar, in numeric form as well as in the visualization of the distributions. To confirm that these patterns hold when controlling for participant-based variation, we also modeled the data using a Bayesian hierarchical model. The model shows largely the same patterns that we see in the descriptive statistics; the credible intervals for all interactions between task and setting cross 0 (i.e., the lower bound of the credible interval is below 0 but the upper bound is above 0). Therefore, the model cannot confidently estimate an effect for setting for any task.

There are four tasks, however, which have slightly larger estimates either in the lab or online. One suggests slightly better scores online: the spelling test. For this, participants were shown 60 words (of which half were spelled incorrectly) and had to indicate which were spelled incorrectly. Online, participants scored an average of 5% better (59% vs. 63%, respectively). The maximum recorded score online was 100% correct, whereas in the lab it was 93% correct. The model also predicts higher scores on the spelling task in the online setting, at the rate of approximately one-third of the standard deviation on the task (0.28 difference between the two settings). While this result may be due to chance (in that better spellers participated in the online setting), it could also be the case that some online participants looked up the spelling of some of words, e.g., on their phones. This would be in line with previous research, which indicates that participants tested online sometimes seek external help in answering challenging questions (Goodman et al., 2013). Importantly, this effect is still minor, although participants were not explicitly discouraged from looking up words.

At the same time, there is little effect of test administration on similar assessments, such as the idiom recognition task. Participants could have just as easily looked up the meaning of an idiom if they were striving to maximize their performance. Thus, we cannot conclude that participants looked answers up whenever possible. Perhaps they only sought external help when it was particularly convenient to do so or when they felt their performance might be judged. For example, being a “bad speller” might be particularly stigmatized compared to not knowing idioms, so participants may have felt compelled to maximize their score on the spelling test only.

Similarly, the digit span task also shows better performance online, where the median is a span of 8 in the lab and 9 online. The model also predicts a difference of 0.18. Here, participants may have used an aid as simple as a pen and paper to jot down numbers. If this is the case, though, it is interesting that the backward digit span does not show a similar trend; the scores on this task do not differ much between settings. This is despite the fact that the backward digit span directly followed the forward digit span. Thus, we cannot say if participants were seeking external aid in this task or if this is simply a spurious result.

On the other hand, two tasks show a trend toward better performance in the lab: antonym production and nonverbal reasoning. Nonverbal reasoning was assessed with Raven’s advanced progressive matrices, known to be a challenging task. Participants must look at an example image, try to deduce the pattern they are seeing, then select the correct continuation of the pattern from eight potential items. We know from previous research that difficult tasks lead to higher dropout rates in online tests (Dandurand et al., 2008). In the current study, dropping out would have reduced participants’ monetary compensation, which likely encouraged them to complete all tests. Thus, they may have underperformed upon losing motivation for the (20-min long) task. In this task, participants in the lab performed about 3% better in terms of mean scores (60% and 57%, respectively). This is also reflected in the model, with a difference of 0.17 between the two settings.

We see a similar trend in the antonym production task, though the reason behind this is less clear. Here, the mean scores are similar (80% correct in the lab and 78% correct online), as are the median and maximum scores. The minimum scores, however, differ by nearly 20% (52% in the lab and 36% online). This means that at least one participant online only correctly named the antonym to a third of the trials. Perhaps this task was also perceived as difficult or annoying by some participants. Taken together with performance on the nonverbal reasoning task, these results play into recent discussions of motivation in psycholinguistic research, which suggest that a portion of data is collected from participants who are bored and disengaged (Christianson et al., 2022). In individual differences research, we often operate under the assumption that participants are at peak performance, showcasing their full abilities. Disengagement with the task could be self-selecting to participants with certain personality characteristics, and thus there could be an extraneous variable in play. However, our results suggest that the impact of this factor is minor and restricted to only some tasks.

Overall, the data hint at two major issues that are surely relevant to individual differences in an online lab: the ability to look up answers online and the likely impact of motivation in a harder task. These two forces have opposite effects: one improves performance online and the other worsens it. However, we have to keep in mind that these differences are small, and the model cannot reliably distinguish them from 0, despite the large sample size. The majority of tasks show no remarkable effect in either direction. The performance online and in the lab is similar, both descriptively and in the model output.

These conclusions are based on a particularly large dataset, with more than 500 participants online. The question thus arises whether the conclusions would have looked the same with a sample size that is more typical for individual differences research and if comparing groups of the same size. In a follow-up step, we fit the model to all in-lab participants (*N* = 149) and a randomly selected subset of online participants (*N* = 149). Naturally, using a smaller dataset increases the amount of uncertainty in the model, which can be seen in the prediction plot (Fig. 6). However, the overall pattern of effects still holds in both subsets, and all tasks except for the spelling test have overlapping credible intervals. The only notable deviation is that the antonym production task does not look as skewed towards higher scores in the online condition in this subset compared to the predictions for the full dataset. This suggests that the model output is generalizable rather than dependent on the unequal group sizes. Nonetheless, we refrain from making sample size suggestions for future individual differences research conducted online since statistical power will depend not only on the number of participants but also on the size of the effect of interest, the number of items in a test, and the type of analysis planned.

In order to go beyond an analysis of group-level means and trends between the two groups, we additionally analyzed the correlational patterns between the test scores and the overarching constructs they were designed to measure. We establish measurement invariance across the settings, which suggests that the psychometric properties of the tests perform the same in both online and in-lab participants (Putnick & Bornstein, 2016). In line with the conclusions we can draw from the model, there are two tests that cause difficulty for full measurement invariance: spelling and antonym production. The fact that the spelling test is not performing the same way across the participant groups seems clear, as it is the test that is most strongly differentiated in performance between the in-lab and the online participants. The error bars do not overlap in the predictions of the Bayesian mixed model and the difference can already be seen in the descriptive statistics. Antonym production was also identified as a test that differed in the two settings; we speculated that this could also be driven by motivation. If these two tasks are allowed to vary between the groups, we can establish measurement invariance for the test battery in regard to participation in the lab or online. This serves as additional evidence for the fact that most of our individual differences tests were unaffected by whether they were presented online or in a laboratory setting.

Given the conclusions from both the mixed model and the measurement invariance analysis, we can make some recommendations for future research. Specifically, we recommend that participant motivation not be overlooked. One way of keeping participants motivated, even for harder tasks, might be to “gamify” the tasks, by making them visually appealing and offering symbolic rewards for peak performance (Christianson et al., 2022; Pedersen et al., 2023). We also recommend paying close attention to the clarity and wording of the instructions, in particular asking participants not to look up answers and letting them know that they are not expected to know the correct answer to every question.

Importantly, differences suggesting lower motivation in the online group and the use of external help were only found for a small handful of tasks. We assessed fifteen individual differences assessments from the IDLaS-NL dataset, and eleven of them showed very similar results both in the lab and online. These tasks are diverse; they assess processing speed, visual and auditory working memory, word production, vocabulary and knowledge of prescriptive grammar rules. On all these tasks, there is neither a detectable difference in the model nor notable deviations in the descriptive statistics. Additionally, we established (partial) measurement invariance, showing that the psychometric properties of the tests did not differ based on whether participants were online or in the lab, for all but two tasks. Thus, we can confidently support online individual differences research into the domains of word production, domain-general skills, and linguistic experience. There seems good enough reason to suggest venturing forth into the online lab, even for individual differences designs that require precise estimation at the individual level.

## Conclusion

The current study shows that in a large-scale individual differences battery, performance online and in the lab was nearly indistinguishable. This holds over multiple tasks of different linguistic and psychological constructs, including timed and untimed linguistic tasks and domain-general skills. Although experimenters should keep an eye on both a participant’s ability to gain external support for a task (e.g., through looking up answers) and the likelihood that they will lose motivation, online experimentation seems to be reliable and precise enough to support individual differences research.

## Data Availability

The data is available at https://osf.io/2knzc/.

## Footnotes

1 IDLaS-NL is a customizable battery that can be used by any interested researcher to create and run an individual differences battery for speakers of Dutch. For more information, see: www.mpi.nl/idlas-nl and Hintz et al. (2023).

2 Test validity statistics are additionally available here: https://www.mpi.nl/idlas-nl/faq

3 Indicators of good fit are CFI > 0.90, TLI > 0.90, and RMSEA < .08 (Van De Schoot et al., 2012).

4 To prove that the more constrained model is not a worse fit to the data, we are looking for *p *values greater than 0.05, as well as a change of less than .01 in CFI and TLI and a change of less than .015 (Chen, 2007).

## Code availability

R code is available at https://osf.io/2knzc/.

## Declarations

### Open practices statement

The data and materials for all experiments are available at https://osf.io/2knzc/. The experiment was not was preregistered.
